# Dasatinib and Quercetin as Senolytic Drugs Improve Fat Deposition and Exhibit Antifibrotic Effects in the Medaka Metabolic Dysfunction-Associated Steatotic Liver Disease Model

**DOI:** 10.3390/diseases12120317

**Published:** 2024-12-04

**Authors:** Shunta Yakubo, Hiroyuki Abe, Yawen Li, Marina Kudo, Atsushi Kimura, Takuya Wakabayashi, Yusuke Watanabe, Naruhiro Kimura, Toru Setsu, Takeshi Yokoo, Akira Sakamaki, Hiroteru Kamimura, Atsunori Tsuchiya, Kenya Kamimura, Shuji Terai

**Affiliations:** 1 Division of Gastroenterology and Hepatology, Graduate School of Medical and Dental Sciences, Niigata University, Niigata 951-9510, Japan; happy7line@gmail.com (S.Y.); liyawenlyw@163.com (Y.L.); rabu_rna@yahoo.co.jp (M.K.); yo-atsu@castle.ocn.ne.jp (A.K.); waka.8453.taku@gmail.com (T.W.); ywatanabe19840421@med.niigata-u.ac.jp (Y.W.); nkimura@med.niigata-u.ac.jp (N.K.); setsut@med.niigata-u.ac.jp (T.S.); saka-a@med.niigata-u.ac.jp (A.S.); hiroteruk@med.niigata-u.ac.jp (H.K.); atsunori@med.niigata-u.ac.jp (A.T.); kenya-k@med.niigata-u.ac.jp (K.K.); terais@med.niigata-u.ac.jp (S.T.); 2 Department of Gastroenterology, Digestive Disease Hospital, Affiliated Hospital of Zunyi Medical University, Zunyi 563000, China; 3 Department of Preemptive Medicine for Digestive Diseases and Healthy Active Life, Graduate School of Medical and Dental Sciences, Niigata University, Niigata 951-8510, Japan; t-yokoo@med.niigata-u.ac.jp; 4 Department of General Medicine, Niigata University School of Medicine, Niigata 951-8510, Japan

**Keywords:** MASLD, NASH, senescence, senolytic drug, medaka

## Abstract

Metabolic dysfunction-associated steatotic liver disease (MASLD) causes cellular senescence due to oxidative stress, endoplasmic reticulum stress, and ectopic fat deposition in the liver. Recently, dasatinib, an antitumor agent, and quercetin, a dietary supplement, were combined as a senolytic drug to eliminate senescent cells. Thus, this study aimed to examine the effects of dasatinib and quercetin administration on removing senescent cells and their therapeutic effects on MASLD in a medaka MASLD model. Dasatinib and quercetin were administered to a medaka MASLD model, which was fed a high-fat diet by dissolving them in aquarium water. The results revealed that senescent cells in the liver were increased in the HFD group but improved in the treatment group. Hematoxylin and eosin staining also showed that treatment improved fat deposition in hepatocytes. In addition, TGFβ1, a driver factor of fibrosis, was reduced in the treatment group. Dasatinib and quercetin eliminated senescent cells in MASLD, attenuated fat deposition, and suppressed fibrosis gene expression. The results indicate that dasatinib and quercetin as senolytic drugs are novel therapeutic agents that reduce MASLD.

## 1. Introduction

Various factors, such as viruses, alcohol, metabolic dysfunction-associated steatotic liver disease (MASLD), and autoimmune hepatitis, can result in chronic liver diseases, such as cirrhosis, liver failure, and hepatocellular carcinoma, with a poor prognosis. MASLD, one of the causes of chronic liver diseases, is not only highly prevalent in developed countries but is also currently rapidly increasing in developing countries, affecting approximately 1.7 billion people [1]. Further increases are feared in the future as diets change [2].

MASLD is a condition that results in ectopic fat deposition in the liver, affecting the metabolism, and is accompanied by oxidative stress, endoplasmic reticulum stress, and autophagy, resulting in hepatocellular damage [3,4,5]. Its treatment targets the above mechanisms and includes diet, exercise, treatment of glucose metabolism disorders, and lipid disorders. However, no adequate treatment for MASLD has been established for disease control.

Recently, senescent cells have been reportedly associated with the MASLD mechanism. Hayflick et al. reported irreversible mitotic arrest in human fibroblasts after successively passing in a cultured cell line in 1961, which was proposed as cellular senescence [6]. This permanent cell division arrest is not limited to mitotic lifespan, as it is associated with telomere shortening, but can be caused by different effects, including DNA and mitochondrial damage [7]. Therefore, cellular senescence has been reported in diseases like pulmonary fibrosis, cardiovascular diseases, and diabetes ** [8,9,10,11]**. The cells that have ceased to proliferate cause a phenomenon known as the senescence-associated secretory phenotype (SASP). SASP affects the surrounding microenvironment via cytokines and other humoral factors that induce inflammation and carcinogenesis. This is responsible for hepatitis and fibrosis progressions in MASLD [12,13].

Dasatinib and quercetin are among the drugs reported to eliminate senescent cells [14]. This study tested the effects of dasatinib and quercetin, the well-known senolytic drugs, on a medaka (*Oryzias latipes*) MASLD model. The drugs can be dissolved in an aquarium tank for uniform administration to each medaka fish, an aquatic organism.

Using immunohistochemistry, we assessed changes in senescent cells by analyzing the correlation of p21 and gH2AX as senescence markers alongside LMNB1, which inversely correlates and serves as a marker. We conducted a histological analysis of fat deposition to determine the therapeutic effect. We performed quantitative polymerase chain reaction (PCR) to evaluate the expression of key genes related to fibrosis and fat metabolism, including TGFβ1, MMP2, and TIMP2, in medaka. To further explore the underlying mechanisms, we examined macrophage changes, assessing CD68 as a pan-macrophage marker and Ym1 as an anti-inflammatory marker for M2 macrophages. This study indicates that the drugs improve fat deposition and inflammation and have antifibrotic effects in a MASLD model.

## 2. Materials and Methods

### 2.1. Animals and Diets

All animal experiments were conducted in full compliance with the Institutional Animal Care and Use Committee regulations at Niigata University (Niigata, Japan), which approved the study protocol. The NBRP medaka (https://shigen.nig.ac.jp/medaka/ accessed on 1 December 2023) provided the d-rR/NAGOYA strain of medaka fish (strain ID: MT847). All animals received humane care following the criteria outlined in the “Guide for the Care and Use of Laboratory Animals” by the National Academy of Sciences and published by the National Institutes of Health. The aquarium was kept in a 2 L tank with 10 medaka fish per tank. The water temperature was adjusted to 24 °C ± 2 °C, humidity to 50% ± 20%, and lighting from 8:00 to 22:00. The control (Hikari Labo M-450; Kyorin, Hyogo, Japan) and high-fat diet (HFD32; CLEA Japan, Tokyo, Japan) were fed at 20 mg/fish/day for 7 weeks, based on previous MASLD medaka reports [15]. Every 13–20 medaka, both male and female, for the three groups of control, HFD, and treatment were prepared and analyzed.

### 2.2. Treatment with Senolytics

Based on previously reported methods, the drugs were administered to medaka through the digestive tract by adding them into the aquarium so that the drug concentration was maintained and administered uniformly to each individual [15,16,17,18]. Dasatinib (QIAGEN, Hilden, Germany) was dissolved at 50 mg in dimethyl sulfoxide overnight with shaking and mixing. Then, 0.01 mg of quercetin was added to 5 mL of phosphate-buffered saline immediately before administration, mixed well, and used. Dose concentrations were determined by evaluating the toxicity tolerability using the trial test (Supplementary Figure S1A). In the pilot study, the quercetin concentrations were set to 0.1 and 0.01 mg/L based on previous reports [19]. Dasatinib concentrations in the tank were established at three levels, corresponding to the C-max concentration found in human blood during clinical administration. Based on the trial results, the solution concentration was 1.0 mg/L for dasatinib and 0.01 mg/L for quercetin, and the tanks were flushed, changed, and dosed once every 2 days. The period and method of administration were determined according to the drug screening tests conducted with medaka fish reported up to now [16,20]. Following the HFD feeding for 6 weeks, the medaka were administered with these drugs for 7 days.

### 2.3. Pathology and Immunohistochemistry

Histological evaluation was performed as previously reported [16]. Samples were fixed in 10% formalin and embedded in paraffin. Sections were prepared in 10-μm thin sections for staining. Then, this was subjected to hematoxylin and eosin (HE) staining and immunohistochemistry staining (IHC). Fat deposition and inflammation were assessed by HE staining and analyzed using the ImageJ software (version 1.6.0_20; National Institutes of Health, Bethesda, MD, USA), which were previously reported [21]. IHC was performed using rabbit anti-p21 polyclonal antibody (orb3599, Biorbyt, Cambridge, UK), rabbit anti-γH2AX polyclonal antibody (ab11174, Abcam, Cambridge, UK), rabbit anti-LMNB1 polyclonal antibody (ab16048, Abcam, Cambridge, UK), rabbit anti-Ym1 polyclonal antibody (#60130, STEMCELL technology, Vancouver, BC, Canada), rabbit anti-CD68 polyclonal antibody (ab125212, Abcam, Cambridge, UK), Vectastain Elite ABC Rabbit IgG kit (PK-6101; Vector Laboratories, Burlingame, CA, USA), and DAB chromogen tablet (Muto Pure Chemicals, Tokyo, Japan) for IHC.

### 2.4. Gene Expression Analysis

RNA was extracted from the liver tissue using NucleoSpin RNA Plus (Takara, Tokyo, Japan). qScript cDNA SuperMix (Quantabio, Beverly, MA, USA) was used for the reverse transcription of cDNA. Gene expression levels were determined by quantitative PCR using QuantStudio 5 Real-Time PCR (Thermo Fisher Scientific, Waltham, MA, USA) and quantified using SYBR Green. The obtained data were compared with the control group (ΔΔCTΔ) using 2Ct with GAPDH as the reference gene (ΔΔCT). The previously reported primers for GAPDH, Col1a1, Mmp2, Timp2b, Tgfβ1, Pparα, and Pparγ were used [17].

### 2.5. Statistical Analysis

Statistical data analyses were conducted using GraphPad Prism V.9.5.1 (GraphPad Software, San Diego, CA, USA). One-way analysis of variance, followed by Tukey’s multiple comparison tests, was used to analyze the obtained data. Bartlet’s test assessed the equality of variances. The Kolmogorov–Smirnov test evaluated the data distribution. *p* < 0.05 was considered to indicate statistical significance.

## 3. Results

### 3.1. P21, γH2AX, and LMNB1 Findings in Histology

**Figure 1:**
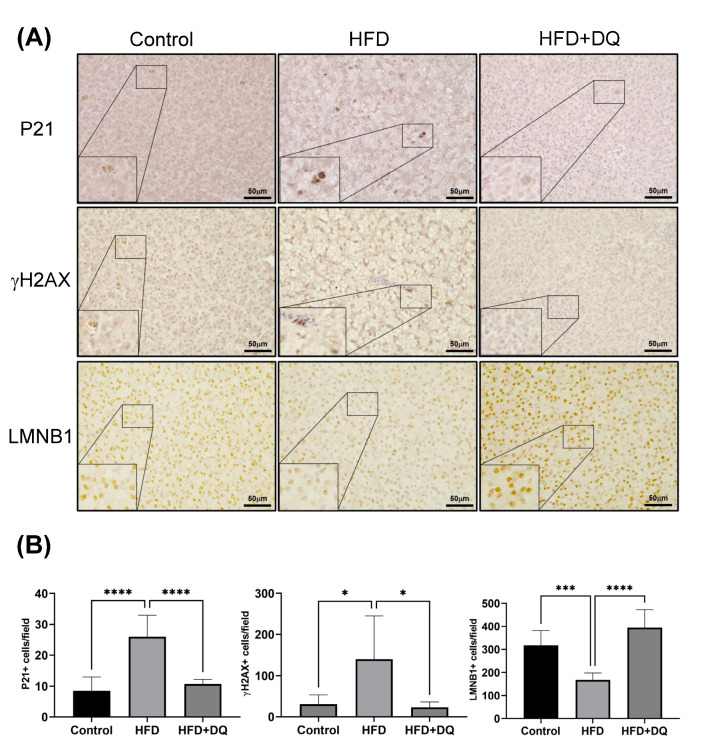
P21, γH2AX, and LMNB1 expressions in liver tissues. (**A**) Representative images of immunohistochemical staining for P21, γH2AX, and LMNB1. (**B**) Quantitative analysis of P21-, γH2AX-, and LMNB1 positive cells. Values are presented as means ± standard deviations. *n* = 13–20 per experiment. * *p* < 0.05, *** *p* < 0.001, and **** *p* < 0.0001.

To evaluate the expression of P21, a marker of senescent cells, the liver tissues of medaka were immunostained for P21 (Figure 1A). The P21-positive cells were higher in the HFD group than the control group (*p* < 0.0001). Conversely, the number of P21-positive cells in the treated group was lower than that in the HFD group and comparable to that in the control group (Figure 1B). IHC for γH2AX, a marker of senescent cells, also revealed decreased positive cells in the treatment group, similar to P21 changes (Figure 1A,B). LMNB1 expression, the loss of which is a senescence-associated biomarker, is decreased in the HFD group compared with the control group (*p* < 0.001). Conversely, the expression in the treatment group improved to the same level as that in the control group (Figure 1A,B).

### 3.2. Macroscopic Findings, Body Weight, and Liver-to-Weight Ratio

**Figure 2:**
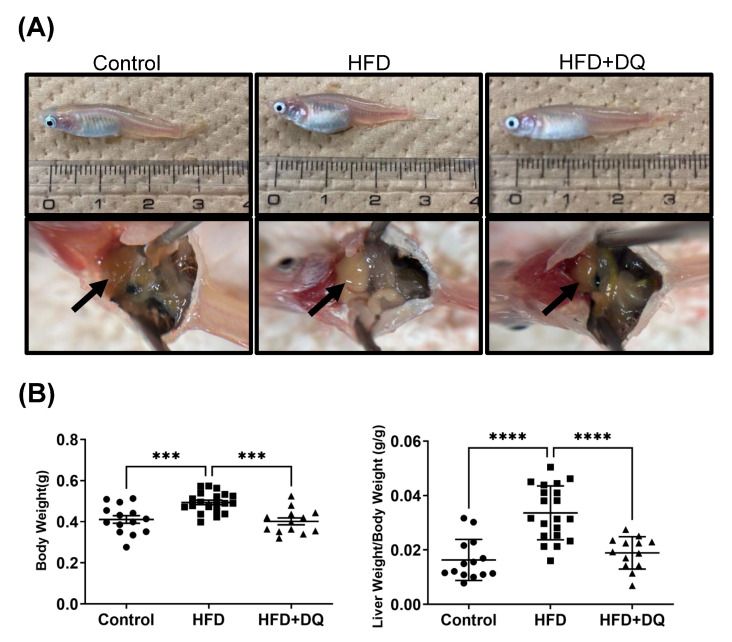
Evaluation of obesity, fatty liver, and hepatomegaly. (**A**) Macroscopic comparison of the body and liver. (**B**) Comparison of the body weight and ratio between the liver and body weights. Values are presented as means ± standard deviations. *n* = 13–20 per experiment. *** *p* < 0.001 and **** *p* < 0.0001.

Figure 2A shows the macroscopic findings of the whole body and liver of the medaka fish under an open abdomen. The whole body of the HFD group showed abdominal distension, and the liver color changed to white with enlarged liver and fat deposition compared with the control group. Conversely, in the treatment group, these findings improved, and the gross findings were similar to those of the control group. Body weights were 0.41 ± 0.07 g, 0.49 ± 0.05 g, and 0.40 ± 0.06 g in the normal diet, HFD, and treatment groups, respectively, indicating an improved body weight in the treatment group, compared with the HFD group (*p* < 0.001). The liver-to-weight ratios were 0.016 ± 0.008, 0.034 ± 0.010, and 0.019 ± 0.006 in the normal diet, HFD, and treatment groups, respectively, indicating an improvement in the treatment group (*p* < 0.01) (Figure 2B). The heights in the HFD and treatment groups did not significantly differ. In addition, the liver size and weight were also increased in the HFD group, reflecting the liver-to-weight ratio, with the treatment group showing improvement to the same level as the control group (Supplementary Figure S2). When evaluating gender differences, the liver-to-weight ratios increased in the HFD group and significantly decreased in the treatment group in both males and females, demonstrating no gender differences (Supplementary Figure S3).

### 3.3. HE Stainings

**Figure 3:**
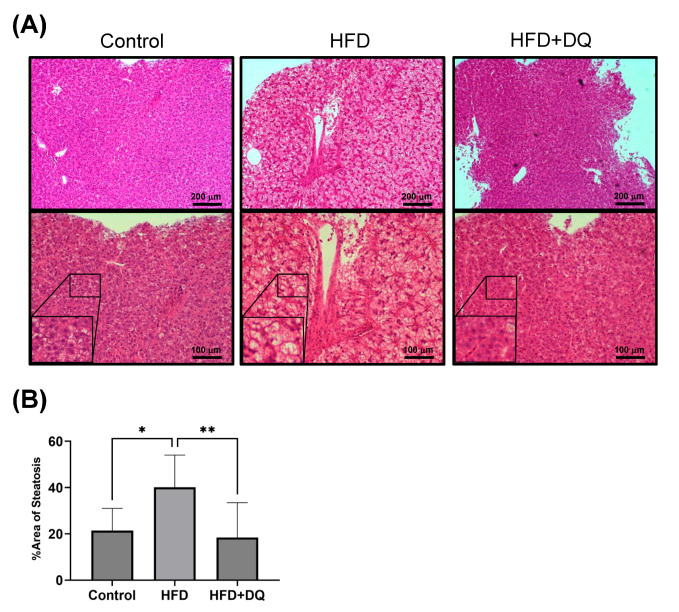
Comparison of fat deposition in liver tissues. (**A**) HE staining of liver tissues. (**B**) Quantitative analysis of fat deposition in liver tissues by HE staining. Values are presented as means ± standard deviations. *n* = 13–20 per experiment. * *p* < 0.05 and ** *p* < 0.01.

Histological fat deposition in the liver was evaluated using HE staining. Histological evaluation revealed that fat deposition in the liver was 21.4% ± 0.6%, 40.1% ± 13.2%, and 18.4% ± 13.9% in the normal diet, HFD, and treatment groups, respectively, indicating improved fat deposition in the treatment group compared with the HFD group (*p* < 0.01) (Figure 3). In addition, inflammatory cell infiltration was prominent in the HFD group.

### 3.4. qPCR Analysis of Fibrosis- and Lipid-Related Gene Expression

**Figure 4:**
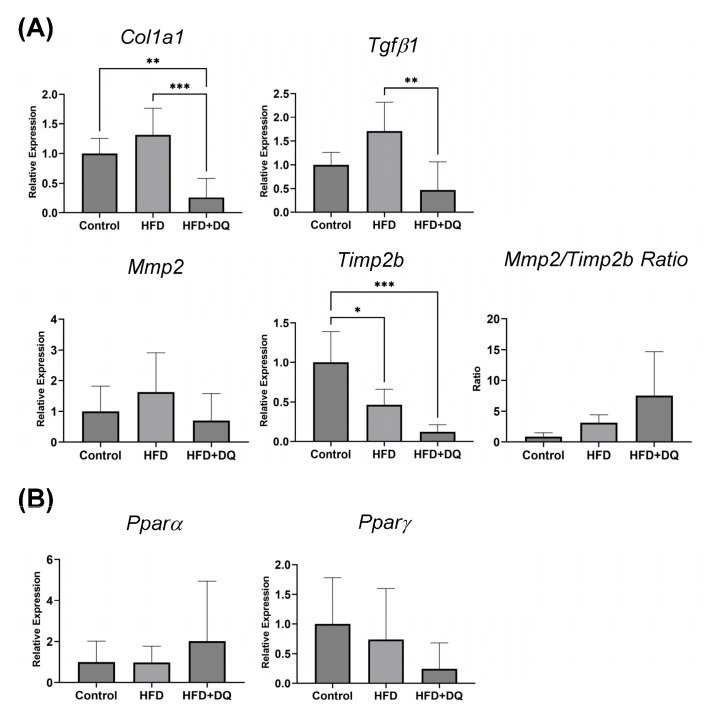
Gene expression analysis in the liver tissue. (**A**) Gene expression of fibrosis-related genes. The analysis focused on the Mmp2-to-Timp2b ratio. (**B**) Gene expression of lipid-related genes. Values are presented as means ± standard deviations. *n* = 13–20 per experiment. * *p* < 0.05, ** *p* < 0.01, and *** *p* < 0.001.

The fibrosis- and lipid-related gene expressions were analyzed by qPCR using liver tissues (Figure 4). The Col1a1, Mmp2, Tgfβ1, and Timp2b expressions were considered fibrosis-related genes. Col1a1 expression was upregulated 1.3 ± 0.4-fold in the HFD group compared with the normal diet group. However, the difference was not significant (*p* = 0.3412), whereas the expression was downregulated 0.26 ± 0.29-fold in the treatment group compared with that in the HFD and normal diet groups (*p* < 0.001). Similarly, Mmp2 expression showed an increasing trend of 1.63 ± 0.75-fold in the HFD group and an improving trend of 0.71 ± 0.79-fold in the treatment group, although the difference was insignificant (*p* = 0.3294). Timp2b expression decreased 0.46 ± 0.17-fold in the HFD group, compared with the normal diet group (*p* < 0.05), and 0.12 ± 0.08-fold in the treatment group, compared with the normal diet group (*p* < 0.001). TGFβ1 was 1.71 ± 0.53-fold higher in the HFD group than that in the normal diet group (*p* = 0.1088) and 0.47 ± 0.54-fold lower in the treatment group than that in the normal diet group (*p* < 0.05). In the treatment group, the Mmp2-to-Timp2b ratio increased in the treatment group compared with the HFD group, but the difference was not statistically significant (*p* = 0.0661).

Regarding lipid metabolism, gene expression of Pparα and Pparγ was confirmed. Although both of them have no significant difference, Pparα showed an increasing trend in the treatment group, whereas Pparγ showed a decreasing trend in both HFD and treatment groups.

### 3.5. Ym1 and CD68 Expressions in Liver Tissues

**Figure 5:**
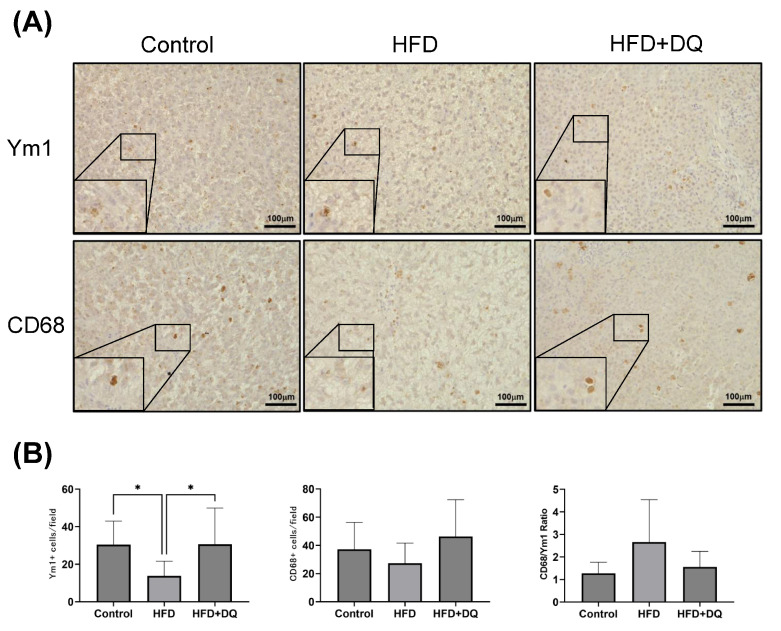
Comparison of M1/M2 macrophages in liver tissues. (**A**) Immunohistochemistry staining for Ym1 and CD68. (**B**) The number of positive cells for Ym1 and CD68 was quantified. The Ym1-to-CD68 ratio was analyzed. Values are presented as means ± standard deviations. *n* = 13–20 per experiment. * *p* < 0.05.

IHC of liver tissues for Ym1 and CD68 was performed to evaluate intrahepatic macrophages (Figure 5). The 30.5 ± 11.6 positive cells of Ym1 per field of view in the control group decreased to 13.8 ± 9.5 in the HFD group (*p* < 0.05) but increased to 30.6 ± 17.9 in the treatment group (*p* < 0.05). The number of CD68-positive cells in the HFD group decreased to 27.3 ± 13.5 compared to 37.2 ± 17.7 in the control group and increased to 46.3 ± 24.3 in the treatment group (*p* = 0.1916).

The CD68-to-Ym1 ratio increased in the HFD group and decreased in the treatment group. The difference was insignificant (*p* = 0.0935).

## 4. Discussion

Senescent cells are increased in MASLD, and cellular senescence of hepatocytes has been reportedly related to glucose metabolism and fibrosis; its effects on lipid metabolism have also been reported [8]. In this study, the improvement of MASLD in a medaka MASLD model, which involves both males and females, was examined by eliminating senescent cells using the senolytic drugs dasatinib and quercetin.

Senolytic drugs induce cell death in senescent cells, with cell cycles being arrested by various mechanisms depending on each drug [22]. Various agents have been reported to eliminate senescent cells, including dasatinib as a single agent, navitoclax, venetoclax, and a two-drug combination of dasatinib and quercetin. Dasatinib removes senescent cells by inducing apoptosis. Conversely, quercetin, a flavonoid derived from plants, exhibits antioxidant and anti-inflammatory properties, suppressing SASP and hindering the survival of senescent cells. They all function as senolytic agents [14].

Additionally, this combination creates a synergistic effect, where quercetin inhibits the survival of senescent cells while dasatinib promotes apoptosis. Since senescent cells exist in diverse environments in vivo, effectively eliminating them with one drug can be challenging. Therefore, this combination therapy produces a more extensive senolytic effect [23]. In addition, quercetin regulates lipid metabolism in its medicinal effect [24]. Quercetin is found in plants and is used as a dietary supplement, whereas dasatinib also has antitumor properties and is used as a therapeutic agent on the clinical side. The use of these two clinically used drugs in the medaka MASLD model, where drugs can be uniformly administered to each fish in a tank, greatly facilitates drug evaluation.

In medaka liver tissues, the expression of P21 and γH2AX, senescent cell markers, was increased in the MASLD model. In contrast, the number of cells expressing these markers was decreased in the dasatinib and quercetin treatment groups. In the MASLD model, these positive cells were not limited to hepatocytes but were also found in other cell types (Figure 1), indicating that dasatinib and quercetin can eliminate various senescent cells in MASLD.

Removal of senescent cells reduced fat deposition in the liver tissue, improved liver weight, and regulated the expression of fibrosis-related genes such as TGFβ1. During the glucose metabolism in the liver, cellular senescence in hepatocytes decreases sensitivity to growth signals and causes insulin resistance [25]. Hepatocyte senescence has also been reported to affect lipid metabolism disorders. Senescent cells switch the energy production to glutamatergic oxidation in glycolysis and mitochondria, leading to impaired energy production. Senescent cells enhanced mTOR signaling and decreased autophagy, affecting lipid metabolism. In addition, this response affects the surrounding microenvironment via SASP. These effects on lipid metabolism induce hepatic inflammation in MASLD [26]. The effects will induce inflammation for macrophages; however, macrophages are also known to accumulate cholesterol in MASLD, causing cellular senescence, and leading to fibrosis induction [27].

In other words, these mechanisms induce inflammation and fibrosis caused by impaired glucose and fat metabolism in senescent cells. In this study, histological evaluation revealed that senescent cell removal reduces fat deposition in the liver. Although no significant difference was observed at the gene expression level, there was a trend toward increased Pparα expression and histologically predominant improvement of fat deposition (Figure 2). This is believed to be due to two factors: senescent cell removal and lipid metabolism improvement by quercetin. Furthermore, this result regulates fibrosis at the gene expression level. Gene expression analysis showed that TGFβ1, a key factor in fibrosis, was suppressed, and the expression of the fibrogenic gene Col1a1 was regulated (Figure 4). We also examined the fibrolytic system associated with fibrosis by assessing Mmp and Timp. We analyzed the gene expression of Mmp2 and Timp2b, which have confirmed expression in medaka. This direct regulation of fibrogenesis, alongside the variations in Timp2b and the Mmp2-to-Timp2b ratio, suggests that the fibrolytic effect is also under regulation.

This includes the effects of the macrophages mentioned above. In this study, the CD68-positive macrophages remained unchanged; however, dasatinib and quercetin treatment increased the anti-inflammatory Ym1-positive macrophages within the liver tissue. The inflammatory macrophages showed no change due to the CD68-to-Ym1 ratio.

These results indicate that dasatinib and quercetin remove senescent cells in the liver in the medaka MASH model and contribute to the improvement of fatty fibrosis. This action decreases the gene expression of TGFβ1, a driver of fibrosis. Furthermore, it was suggested that the mechanism is increased anti-inflammatory macrophages.

Thus, the position of senescent cells in MASLD is important. In addition, Omori et al. reported that senescent cell removal reduces lipidosis and fibrosis in conditionally KO mice that eliminate p16-positive cells [28]. Considering the usefulness of senescent cell elimination in MASLD, senescent cells should be removed by drug therapy, as in this study, to treat MASLD. Furthermore, the paper showed that the efficacy of senescent cell removal in MASH was not affected by gender, suggesting that this drug may be useful for treating MASH without being influenced by sex-related hormones (Supplementary Figure S3).

The limitations of our study include the following: (1) Although fibrosis variation at the genetic level was considered as a limitation in this study, the effect of the SASP factor like IL-6 and IL1b on senescent cells should be evaluated, as the direct effect of their removal because a more advanced fibrosis model was not used (Supplementary Figure S4). (2) As this is an experiment with a single animal model of hepatic steatosis using HFD, additional validation is needed with the safety evaluation of these drugs. In this study, we examined the effects of combining dasatinib and quercetin; however, additional mechanism analysis is necessary. Thus, assessing dasatinib or quercetin individually will help confirm the synergistic effect of the combination therapy. Furthermore, conducting a quantitative evaluation of fat—utilizing Oil Red staining—along with a more thorough histological assessment, investigating fat metabolic pathways, and analyzing changes in the immune system (including macrophages) will help clarify how the elimination of senescent cells is intricately linked to hepatitis and fibrosis.

## 5. Conclusions

Our results suggest that dasatinib and quercetin remove senescent cells, reduce fat deposition in the liver, regulate macrophages and fibrosis-related genes, and improve MASLD in the medaka MASLD model.

## Data Availability

Data will be made available on request.

## Supplementary Materials

The following supporting information can be downloaded at https://www.mdpi.com/article/10.3390/diseases12120317/s1, Figure S1: Toxicity tolerability evaluation of Dasatinib and Quercetin by trial test; Figure S2: The graph of height, liver size, and liver weight; Figure S3: The graph of the ratio between the liver and body weights by gender; Figure S4: Sirius Red staining.
