# Supplementary material for: Dasatinib and Quercetin as Senolytic Drugs Improve Fat Deposition and Exhibit Antifibrotic Effects in the Medaka Metabolic Dysfunction-Associated Steatotic Liver Disease Model
Source: Diseases. 2024 Dec 4;12(12):317. doi: 10.3390/diseases12120317 (PMC11727104; doi:10.3390/diseases12120317)
Supplement: Supplementary file 1 [file diseases-12-00317-s001.zip › Supplementary Figure legends.pdf]

Supplemental Figure Legends

Supplemental Figure S1. Evaluation of toxicity tolerability. Different concentrations of dasatinib and quercetin were administered for 48 hours or for 4 days and toxicity was assessed. n = 5 per experiment.

Supplemental Figure S2. Evaluation of high, liver size, and liver weight. Comparison of the high, liver size, liver weight. Values are presented as means ± standard deviations. n = 13-20 per experiment. *** $p < 0.001$ and **** $p < 0.0001$

Supplemental Figure S3. Evaluation of ratio between the liver and body weights by gender. Comparison of ratio between the liver and body weights by gender. Values are presented as means ± standard deviations. n = 6-10 per experiment. ** $p < 0.01$ and **** $p < 0.0001$

Supplemental Figure S4. Comparison of fibrosis in liver tissues. Sirius Red staining of liver tissues.
