# Supplementary figures and images for: Dasatinib and Quercetin as Senolytic Drugs Improve Fat Deposition and Exhibit Antifibrotic Effects in the Medaka Metabolic Dysfunction-Associated Steatotic Liver Disease Model
Source: Diseases. 2024 Dec 4;12(12):317. doi: 10.3390/diseases12120317 (PMC11727104; doi:10.3390/diseases12120317)

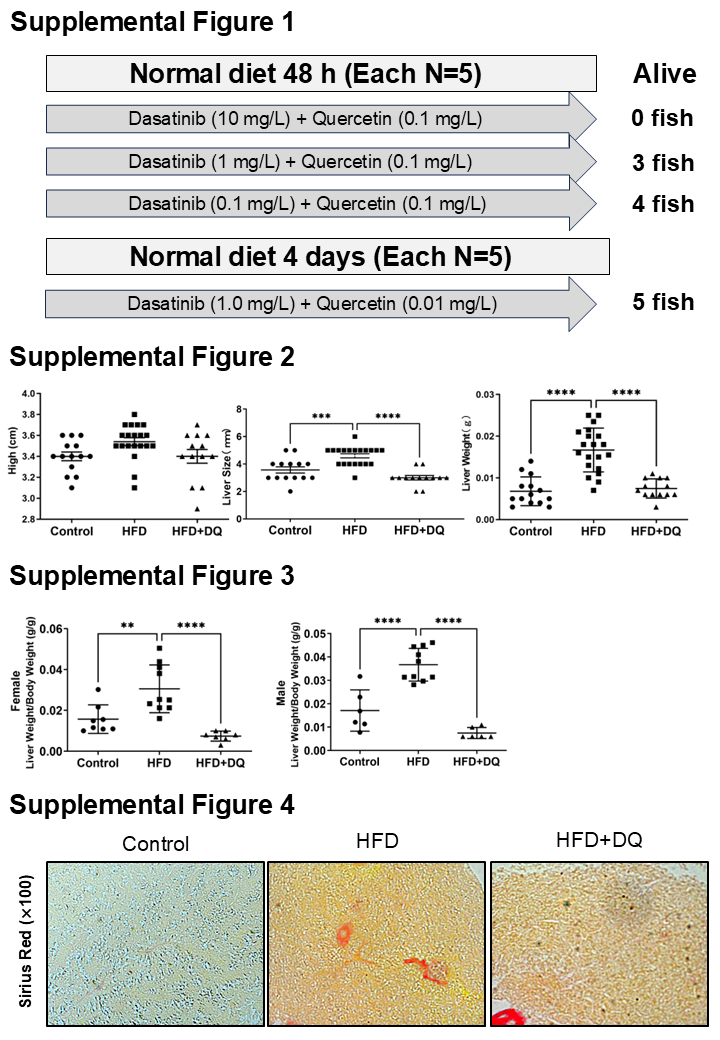

Supplement: Supplementary file 1 [file diseases-12-00317-s001.zip › Supplemental Figures.tif]
